# Single-cell RNA-seq reveals activation of unique gene groups as a consequence of stem cell-parenchymal cell fusion

**DOI:** 10.1038/srep23270

**Published:** 2016-03-21

**Authors:** Brian T. Freeman, Jangwook P. Jung, Brenda M. Ogle

**Affiliations:** 1 Department of Biomedical Engineering, University of Minnesota – Twin Cities, Minneapolis, MN 55455 USA; 2 Stem Cell Institute, University of Minnesota – Twin Cities, Minneapolis, MN 55455 USA; 3 Department of Biomedical Engineering, University of Wisconsin – Madison, Madison, WI 53706 USA; 4 Masonic Cancer Center, University of Minnesota – Twin Cities, Minneapolis, MN 55455 USA; 5 Lillehei Heart Institute, University of Minnesota – Twin Cities, Minneapolis, MN 55455 USA; 6 Institute for Engineering in Medicine, University of Minnesota – Twin Cities, Minneapolis, MN 55455 USA

## Abstract

Fusion of donor mesenchymal stem cells with parenchymal cells of the recipient can occur in the brain, liver, intestine and heart following transplantation. The therapeutic benefit or detriment of resultant hybrids is unknown. Here we sought a global view of phenotypic diversification of mesenchymal stem cell-cardiomyocyte hybrids and associated time course. Using single-cell RNA-seq, we found hybrids consistently increase ribosome components and decrease genes associated with the cell cycle suggesting an increase in protein production and decrease in proliferation to accommodate the fused state. But in the case of most other gene groups, hybrids were individually distinct. In fact, though hybrids can express a transcriptome similar to individual fusion partners, approximately one-third acquired distinct expression profiles in a single day. Some hybrids underwent reprogramming, expressing pluripotency and cardiac precursor genes latent in parental cells and associated with developmental and morphogenic gene groups. Other hybrids expressed genes associated with ontologic cancer sets and two hybrids of separate experimental replicates clustered with breast cancer cells, expressing critical oncogenes and lacking tumor suppressor genes. Rapid transcriptional diversification of this type garners consideration in the context of cellular transplantation to damaged tissues, those with viral infection or other microenvironmental conditions that might promote fusion.

Mesenchymal/multipotent stem/stromal cells (MSCs) can fuse with parenchymal cells of the brain^1^, liver^2^, small intestine^3^ and heart^1^,^4^,^5^,^6^,^7^,^8^,^9^ following transplantation. Fusion of this type might be tightly controlled and restricted to certain cell types as with sperm-egg fusion and skeletal myoblast fusion. However, it is more likely that regulation of fusion is bypassed in the context of transplantation and the altered tissue environment of damaged or diseased tissue. So-called “accidental cell fusion” can result from cell stress including nutrient deprivation and hypoxia which can render cell membranes leaky or unstable^10^,^11^. Unstable cell membranes are biophysically susceptible to membrane fusion^12^. It may be for this reason that cell fusion appears to occur more readily in the context of hypoxia than normoxia^13^,^14^. Accidental cell fusion can also be mediated by viral fusogenic proteins of an active virus or activated elements of an endogenous virus^15^,^16^,^17^,^18^,^19^,^20^,^21^,^22^,^23^. It is estimated that more than 17 of 29 virus families that infect human cells have elements capable of fusing cells^15^.

We and others have proposed that accidental cell fusion can give rise to fusion products capable of acquiring phenotypic and functional properties of either or both fusion partners^2^,^24^,^25^,^26^,^27^,^28^,^29^,^30^,^31^,^32^. The beneficial effects of such an outcome include cell fusion between myeloma cells and B cells to form hybridomas and associated monoclonal antibodies^33^. Similarly, fusion between dendritic cells and tumor cells augments secretion of paracrine factors and can be used as anti-tumor immunotherapy^34^. Return of liver function has been reported after fusion between transplanted bone marrow MSCs and diseased hepatocytes^2^,^25^,^35^ and endogenous c-kit+ cells can form cardiomyocytes in an infarcted murine heart as a result of cell fusion^36^.

Accidental cell fusion might also enable catastrophic events including the development of tumor cells and/or metastatic spread of tumor cells. Spontaneous fusion has been reported between normal breast epithelium and breast cancer cells^37^,^38^, among breast tumor cells themselves^39^,^40^, and between breast cancer epithelium and tumor stromal cells including MSCs^41^,^42^. *In vitro* studies of hybrids formed between normal breast epithelium (M13SV1-EGFP-Neo) and breast cancer cells (HS578T-Hyg) showed increased “locomotory activity” compared to the normal parental line^43^. Fusion-enhanced migration was associated with altered CCL21/CCR7 signaling, which was previously linked to metastatic spreading of breast cancer to lymph nodes. Increased metastatic potential of hybrids was also observed *in vivo* when breast cancer cell variants (MDA-MB-231) with tropism for either lung or bone injected in nude mice gave rise to hybrids capable of metastases to both organs^39^.

Here we probe the extent of transcriptional diversification of hybrids formed between MSCs and cardiomyocytes, and the beneficial or detrimental outcomes of diversification at the single cell level. We probe this particular cell pairing as hybrids of this type have been most frequently reported in the context of cell transplantation to the heart. We utilize single-cell RNA-seq since each hybrid is predicted to be transcriptionally distinct and hence population analyses may mute unique expression profiles.

## Results and Discussion

### Accidental Fusion via Measles Virus Fusogens

**Figure 1:**
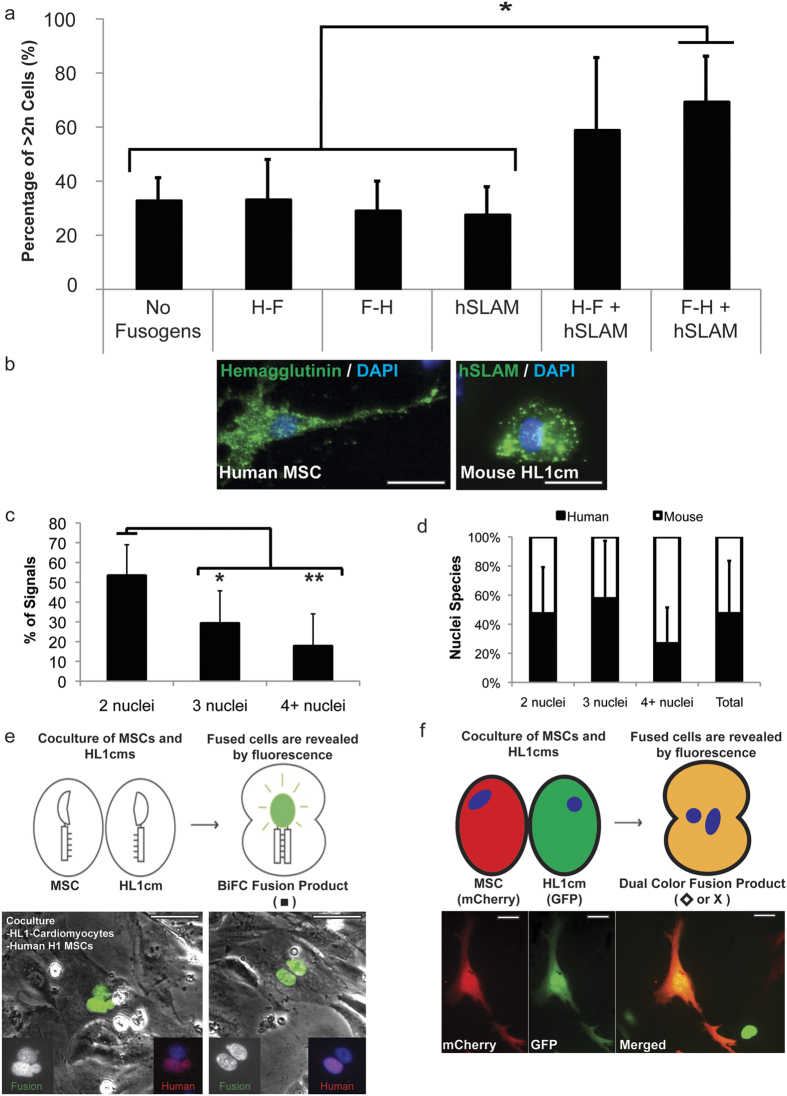
Induction and detection of cell fusion via the measles virus system and bimolecular fluorescence complementation (BiFC) and dual-color fluorescence, respectively. (**a**) DNA content of HL1cm was analyzed via DAPI staining with flow cytometry and DNA content of experimental co-cultures (H–F/hSLAM and F–H/hSLAM) was compared to co-culture controls (no fusogen/no fusogen, H–F/no fusogen, F–H/no fusogen, and hSLAM/no fusogen) (*P < 0.05). Data are represented as mean ± standard deviation (SD). (**b**) Immunocytochemistry for hemagglutinin (H) on hMSC (green) and hSLAM on HL1cm (green). Nuclei stained with DAPI (blue). Scale bar = 50 μm. (**c**) Greater than 50% of detected fusion products contained two nuclei. Data are represented as mean ± SD. (*P < 0.05, **P < 0.01). (**d**) Approximately 50% of the total nuclei of fusion products were of human origin, supporting the specificity of the system. (**e**) Schematic for the bimolecular fluorescence complementation (BiFC) system. Fusion products were detected with fluorescence microscopy for BiFC (green). Cells were then labeled for human nuclear antigen (HNA, red, only present in the hMSC nuclei). Representative fusion products were detected with BiFC (green), HNA (red), and nuclei detected with DAPI (blue). Scale bar = 50 μm. (**f**) Schematic for the dual color fluorescence system. Fusion products were detected with fluorescence microscopy for dual expression of GFP from HL1cm and mCherry from mMSC. Representative fusion product is shown below schematic. Scale bar = 50 μm.

Here we take the case of fusion of MSCs with cardiomyocytes, which has been detected by mulitiple investigators *in vivo*^1^,^4^,^5^,^6^,^7^,^8^,^9^. Single-cell transcriptome analysis of hybrids by RNA-seq should enable direct comparison of individual fusion products with parental cells to determine the degree of programming/reprogramming in hybrid cells toward one or both of the parental cells, the rapidity with which programming/reprogramming occurs and the extent to which unique advantageous or deleterious transcriptome features emerge. To this end we developed a means to efficiently induce accidental cell fusion between MSCs and cardiomyocytes *in vivo*^9^ and *in vitro* (shown here) via expression of viral fusogens. The *in vitro* system mimics the measles virus and associate receptor and enables fusion only when the hemagglutinin (H) protein binds to the human signaling lymphocytic activation molecule (hSLAM), which then forms a trimeric complex with the fusion protein (F) to initiate fusion. To test the specificity of the system, two separate populations of HL-1 cardiomyocytes (HL1cm) were transfected with a bicistronic H–F, bicistronic F–H, hSLAM, or no construct. Co-cultures were generated containing HL1cm transfected with each combination (i.e., H–F/hSLAM, F–H/hSLAM, H–F/no construct, etc.) and monitored for seven days. When all three parts of the fusion system were delivered (either H–F/hSLAM or F–H/hSLAM, Fig. 1a,b), the percentage of cells with DNA content greater than 2n increased from about 30% in controls to 59.1% ± 26.6% (*P* = 0.23) and 69.5% ± 16.7% (^*^P < 0.05) respectively, which confirms the specificity of the system.

To ensure that fusion occurred between two discrete fusion partners, we induced fusion between mouse HL1cm and human H1 MSCs (hMSC) and detected fusion products using a bimolecular complementation (BiFC)-based reporter assay^44^ (Fig. 1e). This technique is a powerful tool for detecting fusion *in vitro* and its use was essential for these studies to ensure that single-cell transcriptomes emerged from *bona fide* fusion products due to the inducible nature of the signal. After identifying fusion products (Fig. 1e), cells were stained for human nuclear antigen (HNA; present on hMSCs and not HL1cm). Over half of the detected fusion products had two nuclei (53.3% ± 15.7%, ^*^P < 0.05 vs. three nuclei and **P < 0.01 vs. 4+ nuclei) (Fig. 1c). Of the fusion products with two nuclei, 47.2% ± 32.0% were of human origin confirming the ability of the fusion system to merge at least one cell of each type (i.e., MSCs and HL1cm) in any given fusion product (Fig. 1d). These data confirm the ability of the measles virus-based system to promote accidental cell fusion between hMSCs and cardiomyocytes and of the BiFC-based detection method to robustly identify hybrids. To ensure that the BiFC complex did not introduce transcriptional bias unrelated to fusion, we also used a dual color (DC) approach wherein different fluorescence reporter genes were expressed in each fusion partner (Fig. 1f).

### Transcriptome Diversification of MSC-Cardiomyocyte Fusion Products

**Figure 2:**
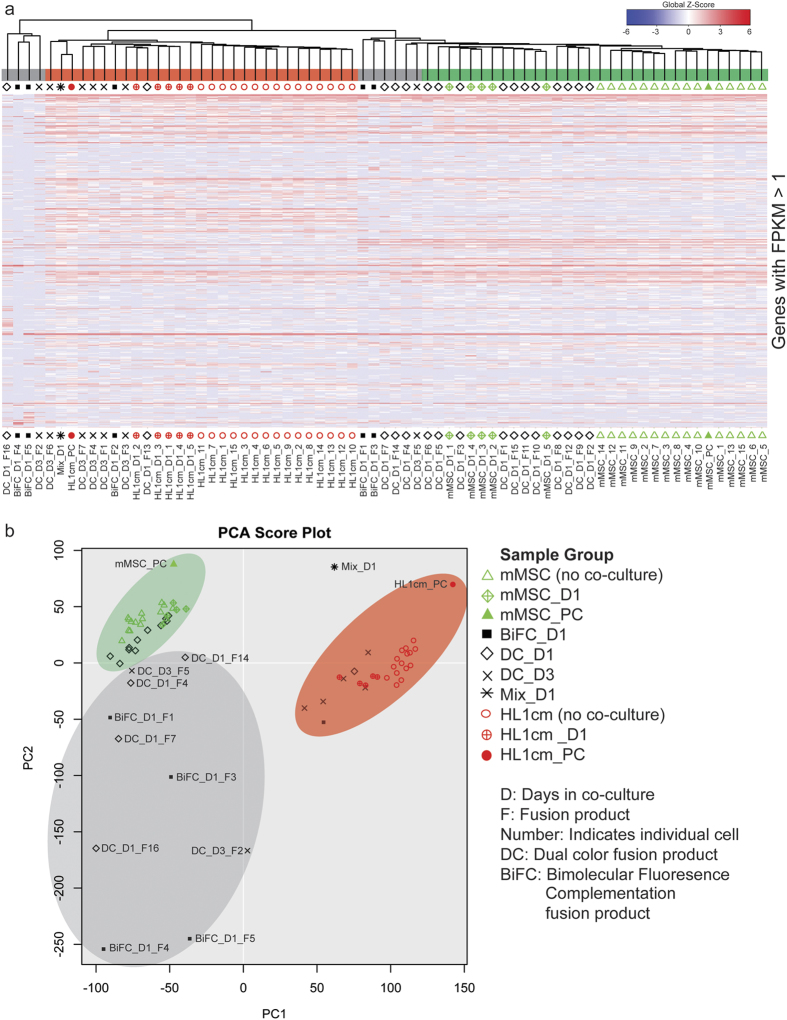
Hierarchical clustering (HC) and principal component analysis (PCA) of all genes (with FPKM >1) of mMSC-cardiomyocyte fusion products and parental controls. (**a**) A global view of gene expression of hybrids (BiFC_D1_F1–5, DC_D1_F1–16, and DC_D3_F1–7), parental cells (mMSC_1–15, mMSC_D1_1–5, HL1cm_1–15, and HL1cm_D1_1–5) and population controls (mMSC_PC, HL1cm_PC, and Mix_D1). Global Z-Score reflects the number of standard deviations away from the mean of expression of all genes in the display. Gene expression is shown in fragments per kilobase of exon per million fragments mapped (FPKM). (**b**) PCA score plot of hybrids, parental cells and population controls.

Single-cell RNA-seq was performed on isolated mouse MSCs (mMSC) HL1cm hybrids. We switched to an autologous murine system as we no longer needed the xenogeneic system to validate the BiFC approach and because most reports of MSC-CM fusion reflect autologous systems^1^,^4^,^8^. Hierarchical clustering (HC) and principal component analysis (PCA) were executed to compare transcripts of five fusion products (BiFC_D1_F1–5, 24 h) identified using BiFC, twenty-three fusion products (DC_D1_F1–16, 24 h; DC_D3_F1–7, 72 h) identified using dual color (DC) expression of GFP and mCherry, the parental controls, and the population controls (mMSC_PC and HL1cm_PC). Parental controls included 15 cells of each parental type isolated prior to co-culture (mMSC_1–15 and HL1cm_1–15) and 5 cells of each parental cell type isolated 24 h after co-culture (mMSC_D1_1–5 and HL1cm_D1_1–5). In addition, a population containing a mixture of both parental cells and fusion products obtained 24 h after co-culture was included (Mix_D1). Resulting HC (Fig. 2a, shown are all genes with Fragments Per Kilobase of transcript per Million mapped reads (FPKM) >1 for any sample^45^) and corresponding PCA plots (Fig. 2b) showed population-level controls correlated with the average of the single cells of that population supporting the accuracy of the single-cell data. The Mix_D1 population control was positioned approximately midway between parental cells reflecting the higher relative fraction of parental cells to fusion products in this population. Analysis of fusion products revealed extensive heterogeneity with ten expressing a unique transcriptome, seven clustering closely with cardiomyocytes and eleven clustering more closely with mMSCs (Fig. 2a,b). Gain or loss of gene expression did not favor particular chromosomes in a substantive way, though minor nuances were noted (Supplementary Fig. S2 and S3).

**Figure 3:**
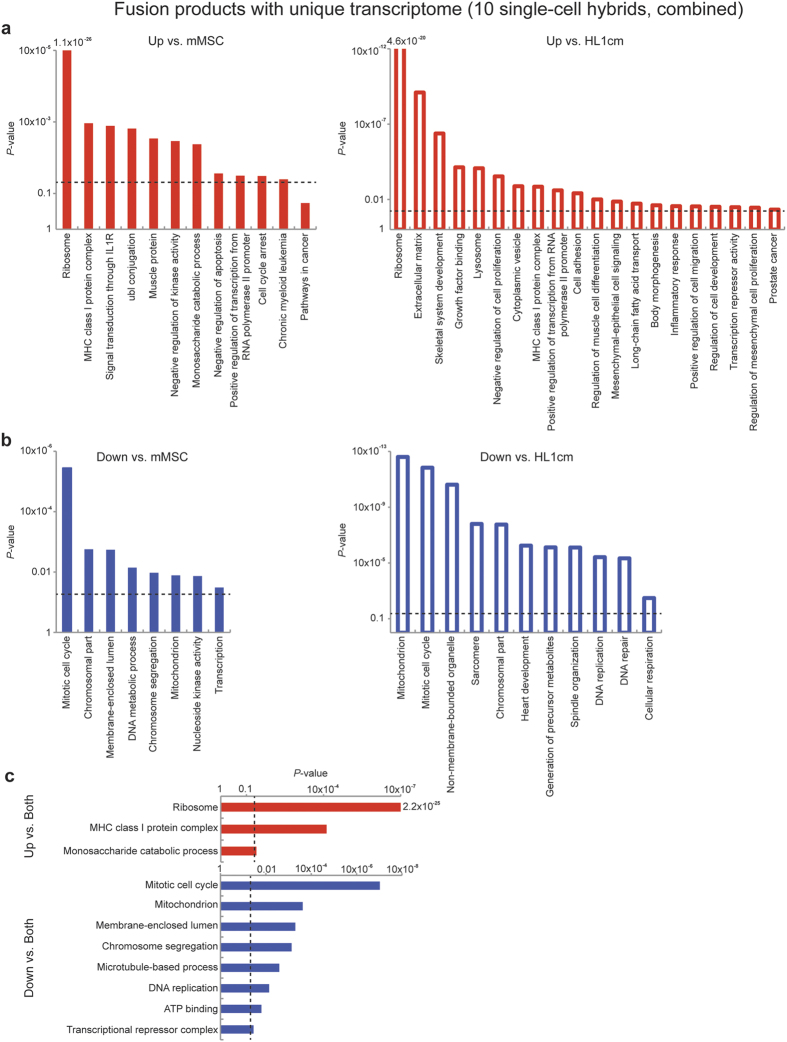
Gene ontology of hybrids with unqiue transcriptomes. *P*-value of functional annotation for differentially expressed genes identified by SCDE for ten hybrids (combined) with unique transcriptomes relative to either fusion partner (see hybrids associated with gray shaded region of Fig. 2a,b). Increased FPKM values are denoted in red (**a**) and decreased FPKM values are denoted in blue (**b**) compared to each fusion partner. Versus HL1cm: open bars, versus mMSC: closed bars. (**c**) *P*-value of functional annotation for differentially expressed genes identified by SCDE for ten hybrids with a unique transcriptome (combined, see hybrids associated with gray shaded region of Fig. 2a,b) relative to both fusion partners (15 of each). Increased FPKM values relative to *both* fusion partners are denoted in red and decreased FPKM values are denoted in blue. The dashed line represents a *P*-value of 0.05. See also Supplementary Table 3.

To identify enriched, function-related gene groups and to isolate interacting proteins of the ten hybrids with unique transcriptomes (combined) relative to parental cells, we used the Database for Annotation, Visualization and Integrated Discovery (DAVID) bioinformatics resources. Significant genes were identified relative to parental controls using the Single Cell Differential Expression (SCDE) toolkit and separated according to increased or decreased FPKM values^46^. From the large list of functional clusters that emerged from our analysis (Supplementary Table S3) certain trends were identified. All hybrids with unique transcriptomes realized a significant increase of ribosomal genes relative to both fusion partners (14 *Rpl* family genes, 9 *Rps* family genes and *Ubb*, Fig. 3a,c). Ribosomal biogenesis is tightly coupled to cell growth and proliferation^47^ and thus we expected to see a corresponding increase in genes associated with the cell cycle. Instead, hybrids with unique transcriptomes exhibited a significant decrease in FPKM values of the genes associated with the mitotic cell cycle, chromosome segregation and DNA replication compared to both fusion partners. Recent studies suggest hyperactive ribosomal biogenesis in the absence of proliferation can be associated with cellular stress including proteotoxic stress, translational infidelity, expression of oncogenes and loss of tumor suppressors^48^. We would add the possibility that cellular stress associated with cell fusion might stimulate ribosomal biogenesis as the cell attempts to recalibrate protein synthesis. Recalibration efforts of the cell might also account for decreased FPKM values associated with non-membrane bounded organelles and membrane enclosed lumen (Fig. 3b). Most other significant changes in gene groups of hybrids occurred relative to one or the other fusion partner. For example, when compared to parental cardiomyocytes the fusion products with unique transcriptomes combined showed a decrease in genes associated with heart development and sarcomere formation suggesting a loss of cardiomyocyte contractile function, which was similar to the results found in an *in vitro* functional study of hybrids formed between human MSCs and neonatal rat ventricular myocytes^32^.

### Function-related Gene Groups of Hybrids Can Mirror Parental Groups

**Figure 4:**
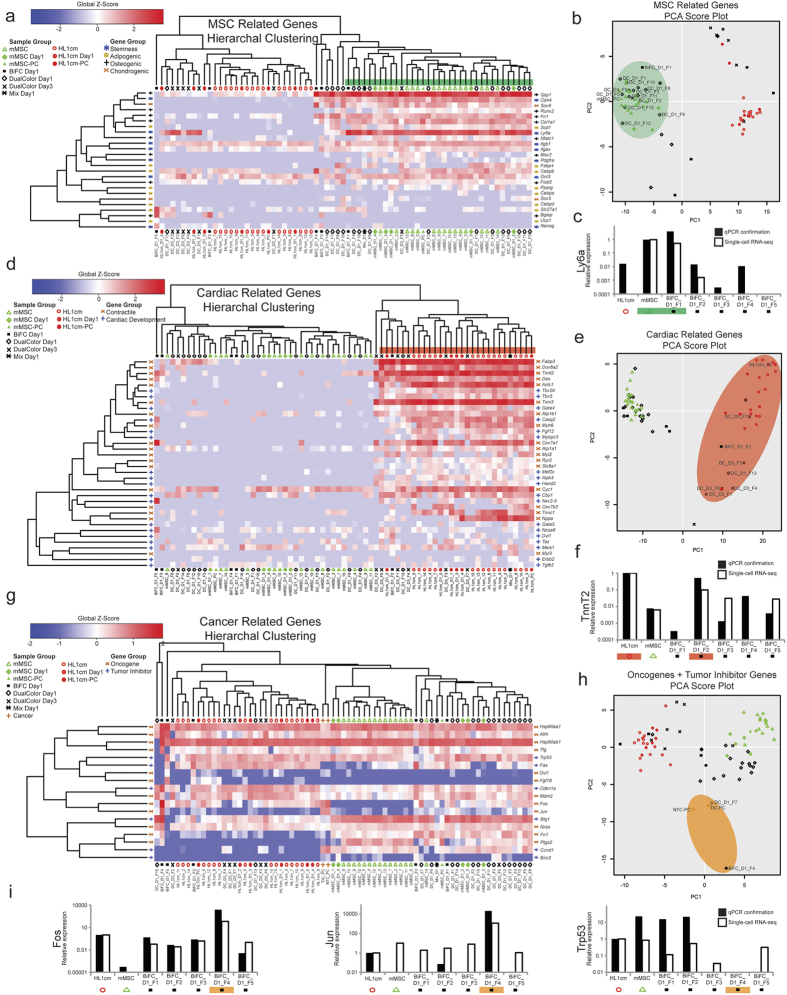
mMSC-cardiomyocyte fusion products can express a cardiomyocyte cell-like, stem cell-like or distinct transcriptome. (**a**) HC of fusion products in relation to a set of genes related to stemness (*), adipogenic differentiation (o), osteogenic differentiation (+), or chondrogenic differentiation (x). (**b**) PCA analysis of fusion products and controls for the MSC gene set. (**d**) HC of fusion products for a set of genes related to cardiac development (+) or contractile ability (x). (**e**) PCA analysis of fusion products and controls for the cardiac gene set. (**g**) HC of fusion products, a population of tumor initiating cancer cells (TIC-PC), a population of nontumorigenic cancer cells (NTC-PC), and controls for a set of oncogenes and a set of tumor suppressor genes. (**h**) PCA analysis of fusion products, cancer populations and controls for the combined oncogene tumor suppressor gene set. (**c,f,i**) qPCR confirmation of RNA-seq data. See also Supplementary Table 4 and Supplementary Fig. 4.

To probe more deeply the contribution of parental lines to programming/reprogramming of hybrid transcriptomes, we defined specific gene sets relevant to each parental cell type including genes related to the differentiation of each parental cell (i.e., mesoderm and cardiac precursors for cardiomyocytes and pluripotent cells for MSCs) and, in the case of MSCs, genes related to mesodermal cell types that can arise from MSCs (i.e., adipocytes, osteoblasts, chondrocytes, Supplementary Table S4). For the MSC gene set, thirteen of twenty-eight hybrids (BiFC_D1_F1, DC_D1_1–5, 8–12, 15, DC_D3_F5) had a profile quite similar to mMSCs (Fig. 4a,b). These thirteen expressed genes related to stemness (*Ly6a, CD44, Itgb1, Itgav*, and *Gnl3*) and mesodermal differentiation (*Spp1, Sox9, Col1a1, Scd1* and *Fn1*). RNA-seq data was confirmed with qPCR analysis of *Ly6a* (Fig. 4c). BiFC_D1_F3 and BiFC_D1_F5 (which do not cluster with mMSCs according to this gene set and clustered with neither mMSC nor HL1cm according to the entire gene set, Fig. 2) expressed high levels of the pluripotency gene, *Nanog* (68.8 and 124.5 FPKM in BiFC_D1_F3 and BiFC_D1_F5 respectively). This was unexpected since neither mMSCs (average 1.92 ± 5.34 FPKM) nor HL1cm (average 3.57 ± 6.03 FPKM) express *Nanog* at a high level. *Nanog* is a transcription factor, known for its control of proliferation and self-renewal in the inner cell mass, supporting a reversion or reprogrammed cell state for BiFC_D1_F3 and BiFC_D1_F5. Of note, twelve of the thirteen hybrids that clustered with mMSCs were obtained one day following co-culture as opposed to those obtained three days after co-culture which either clustered with HL1cm or expressed a unique transcriptome. To explore this trend we next focused on genes associated with cardiomyocyte determination and contractility.

Seven (BIFC_D1_F2, DC_D1_F13, DC_D3_F1, 3, 4, 6, 7) of twenty-eight hybrids had a transcriptome quite similar to HL1cm (Fig. 4d,e). Of the seven, five corresponded to hybrids three days after co-culture. These seven hybrids expressed genes related to cardiac development to a lesser extent (*Hand2*, *Cby1, Tbx5, Tbx20, and Gata4*) and to contractile machinery to a greater extent (*Actc1*, *Tnni3*, *Cox6a2*, *fabp3*, *Cyc1, Atp1a1, Atp1b1, Des*, *Tnnt2*, and *Myh6*). RNA-seq data was confirmed with qPCR analysis (Fig. 4f). Of particular interest, BiFC_D1_F5 (which clustered far from HL1cm according to this gene set and clustered with neither mMSC nor HL1cm according to the entire gene set, Fig. 2), expressed a vastly different cardiac gene profile overall and unexpectedly expressed mesodermal precursor gene *Nkx2.5* (2545 FPKM) at a level far exceeding the cardiomyocyte controls (average *Nkx2–5* expression 12.6 ± 18.2 FPKM) (Fig. 4d). This is consistent with the ontology of hybrids with unique transcriptomes showing increased regulation of muscle cell differentiation when compared to HL1cm (Fig. 3a). Of note, BiFC_D1_F5 also expressed other developmental (*Ncoa6*, *Dvl1*) and contractile genes (*Cox7a1*, *Des*, *Tnnt2* and *Myh6*) at levels comparable to cardiomyocyte controls. Enticing is the possibility that fusion can enable reprogramming to an early mesodermal state although it should be made clear that single-cell sequencing technologies sample only approximately 5–10% of the total transcripts of a cell, and therefore low-abundance genes including transcription factors associated with cell differentiation may have been missed^49^, especially in hybrids where *bona fide* replicates are challenging to obtain and more challenging to confirm.

### Cancer-related Gene Groups Prevalent in Some Hybrids

To this point, analyses of transcriptomes of fusion products have been biased toward beneficial outcomes of fusion. Since ten hybrids expressed dramatically diverse and distinct transcriptional features, we reassessed the ontologic outcomes related to cancer fates. Recall from Fig. 3a, that cancer gene clusters (Chronic myeloid leukemia vs. mMSC and Prostate cancer vs. HL1cm) were identified when compared with both mMSC and HL1cm at statistically significant levels. As neither parental cell type had a cancer-like phenotype, we conducted further analysis on genes in these sets to discern the prevalence (i.e., number of hybrids) and degree of tumor susceptibility of hybrids (i.e., clustering with *bona fide* tumor cells). These genes were classified into one list containing two gene groups, oncogenes or tumor inhibitors (Supplementary Table S4). HC and PCA were performed (Fig. 4g,h, Supplementary Fig. S4). In addition, we included RNA-seq data from two populations (tumor initiating cells (TIC-PC) and nontumorigenic cancer cells (NTC-PC)) of MMTV-*Wnt-1* murine tumor cells (previously reported^50^). We selected these breast cancer cells because they were processed *in silico* in a manner similar to our approach and therefore FPKMs could be directly compared. Preferable may have been prostate or leukemia cells, but we were unable to find an appropriate single-cell RNA-seq data set. Despite this, the PCA plot showed BiFC_D1_F4 and DC_D1_F7 clustered with both TIC-PC and NTC-PC. Of note, BiFC_D1_F4 and DC_D1_F7 showed decreased FPKM values for the tumor suppressor gene, p53 (*Trp53*, no detectable FPKM and 6.65 FPKM, respectively) and increased levels of the proto-oncogenes *Fos* and *Jun*. *Fos* levels were 16952.1 FPKM for BiFC_D1_F4 and 754.0 FPKM for DC_D1_F7. *Jun* levels were 1581.5 FPKM for BiFC_D1_F4 and 14.1 FPKM for DC_D1_F7. Average oncogene levels for parental cell with highest expression (*Fos*, 128.2 ± 111.2 FPKM, HL1cm and *Jun* 3.49 ± 3.62 FPKM, mMSC) were less than levels observed in these two hybrids and *Trp53* expression (231.5 ± 165.8 FPKM, mMSC; 232.1 ± 144.7 FPKM HL1cm) is reliably detected as opposed to undetected/low levels of these two hybrids. These RNA-seq trends were confirmed with qPCR analysis of *Fos, Jun*, and *Trp53* (Fig. 4i). BiFC_D1_F4 even had higher levels of *Fos* (550.3 and 1201.5 FPKM for TIC-PC and NTC-PC, respectively) and *Jun* (421.1 and 477.0 FPKM for TIC-PC and NTC-PC, respectively) expression than the breast cancer cell populations (accounting in large part for the spread between BiFC_D1_F4 and the TIC/NTC in the PCA score plot, Fig. 4h, Supplementary Fig. S4). Thus the increased FPKM values of oncogenes, clustering with tumor-forming cancer cell populations, and decreased or undetected FPKM values of tumor suppressors in some fusion products suggests transcriptome diversity associated with accidental cell fusion has potential to support the emergence of a cancer phenotype.

## Conclusions

Thus, here we utilize a robust means to increase accidental cell fusion *in vitro* and an inducible detection system to reliably identify and isolate fusion products subsequently analyzed using single-cell RNA-seq. We found ten hybrids expressed a unique transcriptome, seven clustered closely with cardiomyocytes and eleven clustered more closely with mMSCs supporting the hypothesis that cell fusion is an ingenious means to generate transcriptional diversity. Grouping of hybrids might reflect, in part, the ratio of parental nuclei^51^. Figure 1 indicates approximately 47% of hybrids have more than two nuclei and interestingly 64% of hybrids showed significant similarity to one or the other fusion partner. However, at the later time point (day 3) hybrids appear to tend toward a cardiomyocyte-like phenotype compared to day 1 (Chi-square statistic, 10.73; *P* < 0.005 of HC, Fig. 2a) suggesting such hybrids are more apt to survive or that numbers of nuclei alone do not dictate phenotypic outcomes. Gene ontology analysis of differentially expressed genes showed that the fusion products with unique transcriptomes consistently recalibrate protein synthesis and limit proliferation as indicated by increased FPKM values of genes associated with ribosomal proteins and decreased FPKM values of genes associated with cytoplasmic organelles and the cell cycle, respectively. Some fusion products underwent considerable reprogramming expressing pluripotency and cardiac precursor genes at high levels relative to parental cells. Also of interest was the observation that two hybrids appeared to produce a transcriptome, which aligned closely to that of breast cancer cells, increasing critical oncogenes and decreasing tumor suppressor genes. For these reasons, it will be important to monitor stem cell transplantation and other scenarios that enable membrane instability for the emergence of tumors.

## Methods

Human MSCs (hMSC), mouse bone marrow MSCs (mMSC) and mouse HL-1 cardiomyocytes (HL1cm) were expanded and cultured as previously described^52^,^53^. hMSCs were used only in Fig. 1 to confirm specificity of the measles virus system. mMSCs were used for single-cell RNA-seq experiments. The first five fusion products were detected via the inducible bimolecular fluorescence complementation (BiFC) system^44^. The remaining twenty-three hybrids were detected via the more commonly used dual-color (DC) fluorescence methodology. Briefly, GFP was constitutively expressed in HL1cms and mCherry in mMSCs. Hybrids in this case are dual-labeled with both GFP and mCherry. BiFC positive (GFP) cells or dual-labeled cells were sorted using FACS. Sorted, single-cell, fusion products were captured on chips using the Fluidigm C1 system. mRNA libraries were constructed using the Illumina Nextera XT according to the manufacturer’s protocol and sequenced on the Illumina Miseqv3 (Supplementary Fig. S1, Supplementary Tables S1,S2,S3,S4). RNA-seq data has been uploaded to the Gene Expression Omnibus (GEO) database (GSE69926). RNA-seq data was confirmed with qPCR analysis on the cDNA from the Fluidigm C1 chip for selected genes.

A detailed methods section is available in the Online Data Supplement.

## Additional Information

**How to cite this article**: Freeman, B. T. *et al.* Single-cell RNA-seq reveals activation of unique gene groups as a consequence of stem cell-parenchymal cell fusion. *Sci. Rep.* **6**, 23270; doi: 10.1038/srep23270 (2016).

## Supplementary Material

Supplementary Information

Supplementary Table S1

Supplementary Table S2

Supplementary Table S3

Supplementary Table S4
