# Supplementary material for: Single-cell RNA-seq reveals activation of unique gene groups as a consequence of stem cell-parenchymal cell fusion
Source: Sci Rep. 2016 Mar 21;6:23270. doi: 10.1038/srep23270 (PMC4800419; doi:10.1038/srep23270)
Supplement: Supplementary Information [file srep23270-s1.pdf]

Single-cell RNA-seq reveals activation of unique gene groups as a consequence of stem cell-parenchymal cell fusion

Brian T. Freeman[1,2,3], Jangwook P. Jung,[1,2] and Brenda M. Ogle[1,2,3,4,5,6*]

Author Affiliation:

1. Department of Biomedical Engineering, University of Minnesota – Twin Cities, Minneapolis, MN 55455 USA
2. Stem Cell Institute, University of Minnesota – Twin Cities, Minneapolis, MN 55455 USA
3. Department of Biomedical Engineering, University of Wisconsin – Madison, Madison, WI 53706 USA
4. Masonic Cancer Center, University of Minnesota – Twin Cities, Minneapolis, MN 55455 USA
5. Lillehei Heart Institute, University of Minnesota – Twin Cities, Minneapolis, MN 55455 USA
6. Institute for Engineering in Medicine, University of Minnesota – Twin Cities, Minneapolis, MN 55455 USA

SUPPLEMENTARY INFORMATION

Detailed Methods

Cell Culture

Human MSCs derived from human embryonic stem cells (hMSCs from WA-01), a gift of Dr. Peiman Hematti (University of Wisconsin-Madison) were expanded and cultured as previously described[1]. Also, mouse bone marrow mesenchymal stem cells (mMSCs) were purchased (3 month-old male C57BL mice, Georgia Reagents University, Augusta, GA[2]) and expanded and cultured with the same protocol as the hMSCs. Briefly, MSCs were cultured on a 0.1% gelatin (Sigma Aldrich, St. Louis, MO) pretreated flask containing α-minimum essential medium (MEM) complete. Alpha-MEM-complete consisted of α-MEM (Invitrogen, Carlsbad, CA), 10% fetal bovine serum (Hyclone, Logan, UT), 0.1 mM nonessential amino acids (Invitrogen), and 2 mM L-glutamine (Invitrogren). MSC cultures were allowed to grow to 60-70% confluence and were replated at a concentration of 1,500 cells/cm^2. HL-1 cardiomyocytes (HL1cm) (a gift of Dr. William Claycomb) were also expanded and cultured as previously described[3]. HL1cms were cultured on fibronectin/gelatin (1.25 mg fibronectin/100 mL 0.02% gelatin) (Sigma Aldrich) pretreated flasks containing Claycomb-complete. Claycomb-complete medium was comprised of Claycomb medium (SAFC Biosciences, St. Louis, MO, USA), 10% fetal bovine serum qualified for CMs (SAFC Biosciences), 100 U/mL: 100 μg/mL penicillin-streptomycin (Lonza, Walkersville, MD, USA), 0.1 mM norepinephrine (Sigma-Aldrich), and 2 mM L-glutamine (Invitrogen). HL1cms were passaged at 100% confluence and split 1:3. HL1cm coating and HL1cm medium was used when the two cell types were co-cultured. Experiments were performed using passages 6 – 11 and 70 – 85 for MSCs and HL1cms, respectively. All cultures were maintained at 37°C in 5% CO_2. hMSCs were used only in Figure 1 to confirm specificity of the measles virus system. mMSCs were used for single-cell RNA-seq experiments.

Cell Fusion Induction and Detection of Fusion

To induce "accidental cell fusion" between MSCs (hMSCs or mMSCs) and HL1cms, we utilized the measles virus to create a recombinant DNA fusion system. The recombinant DNA system contains three components and enables fusion only when the hemagglutinin (H) protein of the virus binds to the human signaling lymphocytic activation molecule (hSLAM) of the human cell, which then forms a trimeric complex with the fusion protein (F) of the virus to initiate fusion[4]. HL1cms are transfected with the receptor component, hSLAM, while MSCs are transfected with the hemagglutinin (H) and fusion (F) proteins (viral fusogens provided by Yoshihiro Kawaoka of the University of Wisconsin-Madison). Fusion only occurs when all three proteins are present and we utilized this ability to induce fusion between MSCs and HL1cms (**Figure 1a**). The first five fusion products (BiFC_D1_F1-5) were detected via the inducible bimolecular fluorescence complementation (BiFC) system [5]**.** In this system, fluorescence will only be seen in cells that express both the VNH3.1 and YCH3.1 proteins, which are both encoded on separate plasmids and are linked to histones so that the fluorescence will occur in the nucleus. In our system the MSCs were given the YCH3.1 protein and HL1cms were given the VNH3.1 protein, so fluorescence will be detected only after fusion between MSCs and HL1cms. The remaining twenty-three hybrids (DC_D1_F1-16 and DC_D3_F1-7) were detected via the more commonly used two-color fluorescence methodology. Briefly, GFP was constitutively expressed in HL1 cardiomyocytes and mCherry in mMSCs via transfection. Hybrids in this case are dual-labeled with both GFP and mCherry after the HL1cm and mMSC share cytoplasm following fusion. Importantly there were five putative fusion products isolated using the dual color system that were deemed false-positive based on punctate fluorescence of one fluorophore or the other indicating likely endocytosis of cell debris and not bona fide fusion. These five false positive were not included in this manuscript**.** Transfection was accomplished using the Neon Transfection System (Invitrogen, Carlsbad, CA), according to the manufacturer's protocol. Briefly, 5 x 10^5 HL1cms were transfected with 2 μg of hSLAM and 2 μg of the detection system plasmid (VNH3.1 BiFC or pCAGS-GFP) with one 1,300 V pulse for 30 msec and plated into 6-well plates (Falcon, Fisher Scientific, Forest Lawn, NJ) containing Claycomb-complete medium without penicillin-streptomycin as per Neon Transfection System protocol. The HL1cm electroporation was repeated and added to the same well to obtain approximately 1 x 10^6 total cells transfected. Eighteen hours later, fresh Claycomb-complete medium without penicillin-streptomycin was added to the transfected HL1cm and 5 x 10^5 MSCs were transfected with 2 μg of F-H and 2 μg of the detection system plasmid (YCH3.1 BiFC plasmid or pCAGS-GFP) with one 1,500 V pulse for 20 msec and plated directly onto the previously electroporated HL1cms in the 6-well plate. The co-culture was allowed to incubate overnight and the fusion products were analyzed on the following day and identified using fluorescence microscopy for GFP.

Immunocytochemistry

To confirm delivery of the vectors as well as detection of human nuclei, electroporated hMSCs and HL1cms were plated for 24 hours and immunocytochemistry (ICC) was performed to detect the H protein in MSCs, the hSLAM protein in HL1cms, or human nuclei in fusion products. We did not probe for the F protein because of the bicistronic nature of the F-H construct. Briefly, cells were washed with two rinses and two incubations of 1X PBS. Cell fixation was performed with 4% PFA for 15 min, followed by another set of washes, and probed with the 1:25 dilution of mouse anti-hemagglutinin antibody (35-614, ProSci Incorporated, Poway, CA), mouse anti-hSLAM antibody (ab2604, Abcam, Cambridge, MA) or mouse anti-human nuclear antigen (MAB1281, Millipore, Temecula, CA) in a dilution buffer of 5% bovine serum albumin (Hyclone, Logan, UT), 2% goat serum (MP Biomedical, Solon, OH), 1% glycine (Sigma Aldrich, St. Louis, MO), and 0.1% triton-X (MP Biomedical, Solon, OH). Forty microliters of this antibody solution were placed on fixed co-cultures overnight at 4°C. Sections were washed with 1X PBS and

incubated for 45 minutes at 4°C with 40 µL of a 1:200 dilution of the secondary antibody (AF647 donkey anti-mouse, Invitrogen, Carlsbad, CA) in dilution buffer. Cultures were washed a final time and mounted in DABCO/DAPI mounting medium (2.5% 1,4-diazabicyclo[2.2.2]octane (Sigma-Aldrich), 50% glycerol (Fisher Scientific, Forest Lawn, NJ, USA), and 0.005% 4′,6-diamidino-2-phenylindole (Sigma-Aldrich) in PBS). Fluorescence emission was detected on an IX71 inverted deconvolution fluorescence microscope (Olympus, Center Valley, PA). Images were acquired with a 20X UPlanFluor objective (NA = 0.5), using Metamorph software (Molecular Devices, Sunnyvale, CA) and analyzed with ImageJ (Fiji; open source software, http://pacific.mpi-cbg.de/wiki/index.php/Fiji). Background fluorescence was determined using a secondary antibody only control.

Flow Cytometry Analysis

To test the specificity of the system, two separate populations of HL-1 cardiomyocytes were transfected with the bicistronic H-F, bicistronic F-H, hSLAM, or no construct. Co-cultures were generated containing HL-1 cardiomyocytes transfected with each combination and monitored for seven days. Transfected HL-1-cardiomyocytes were trypsinized (0.25% trypsin, Mediatech, Inc. Manassas, VA) and fixed with 4% paraformaldehye in suspension after seven days in culture. The DNA content was analyzed via DAPI staining (0.005% 4',6-diamidino-2-phenylindole in PBS) with flow cytometry and DNA content of experimental co-cultures (H-F/hSLAM and F-H/hSLAM) was compared to coculture controls (no fusogen/no fusogen, H-F/no fusogen, F-H/no fusogen, and hSLAM/no fusogen). The unfused cells should have normal diploid (2n) DNA content, but any cell that is undergoing division or has fused will result in DNA content greater than 2n. The percent of cells greater than 2n was quantified by setting a threshold past the 2n peak in the negative control histogram.

Single-cell Capture and RNA-seq

For the first five fusion products isolated (BiFC_D1_F1-5), co-cultures of transfected mMSCs and HL1cms were trypsinized after 18 h and suspended in 1X PBS. For the twenty-three dual color hybrids isolated, co-cultures of transfected mMSCs and HL1cms were trypsinized after 18 h (DC_D1_F1-16) or 72 h (DC_D3_F1-7) and suspended in 1X PBS. BiFC positive (GFP^{+}) or dual color positive cells (GFP^{+} and mCherry^{+}) were sorted using FACS (Aria II, BD Biosciences) into Claycomb-complete medium in a 6-well plate**.** The sorted cells were imaged with phase-contrast and fluorescent microscopy to confirm the BiFC or dual color signal. The sorted cells (approximately 300-1000 cells) were centrifuged and resuspended in 5 µL of Claycomb-complete medium for addition to the capture chip. Sorted, single-cell, BiFC-positive or dual color-positive, fusion products were captured on a large-sized (17-25 µm cell diameter) chip using the Fluidigm C1 system. Cells were loaded into the chip at approximately 20-50 cells/µL. The BiFC fusion products were stained for viability (DEAD cell viability assay; Molecular Probes, Life Technologies, Grand Island, NY). Captured fusion products were imaged with phase-contrast and fluorescence microscopy to confirm cell number, viability and BiFC or dual color positivity at each capture point. Only single, live, BiFC or dual color positive cells were included in the fusion analysis. mMSCs and HL1cms controls without co-culture were captured in a separate Fluidigm C1 device with the HL1cm labled with a green cytoplasmic dye (1 µm CellTracker Green CMFDA, Molecular Probes, Eugene, OR). Fifteen single, live cells of each control were selected for analysis (mMSC_1-15 and HL1cm_1-15). Also, five mMSCs and five HL1cms that were captured in the same chip as the dual color day one fusion products and were selected for analysis (mMSC_D1_1-5 and HL1cm_D1_1-5). These ten controls underwent co-culture for 24 h after transfection without fusing**.** Once cells were captured in the device, cDNAs were prepared from each cell on the chip using the SMARTer Ultra Low RNA kit for Fluidigm C1 System (Clontech, Mountain View, CA). RNA spike-in Mix (Ambion, Life

Technologies) was added to the lyses reaction and processed along side to cellular mRNA. mRNA library was constructed using the Illumina Nextera XT preparation kit (Illumina, San Diego, CA) according to the manufacturer's protocol and sequenced on the Illumina MiSeqv3 using paired end reads with a length of 75 bp to a depth of 18 to 22 million reads with the Multiplex on one MiSeq lane to create *.fastq files. For each experiment, a bulk population RNA control of both mMSCs and HL1cm was run in parallel to the single-cell samples. In addition, a population containing a mixture of both parental cells and fusion products obtained 24 hours after co-culture was included (Mix_D1). Single-cell capture, cDNA preparation and RNA-seq was performed with the help of Kenneth Beckman, Adam Hauge and Jerry Daniel of the University of Minnesota Genomics Center.

Gene Expression Analysis

Gene expression analysis was performed with Galaxy software (Minnesota Supercomputing Institute (MSI), University of Minnesota, Minneapolis, MN). Reads were processed and aligned to the mouse reference genome (mm10_genes_2012_05_23.gtf and canonical_mm10.fa) using Tophat (version 2.0.12, open source software, http://ccb.jhu.edu/software/tophat/index.shtml)[6]. See Table S1 for information about the total number of reads and percent concordant mapped reads for each cell. The default options supplied with the software were used and the aligned read files produced by Tophat were processed using Cufflinks software (version 2.2.1, open source software, http://cole-trapnell-lab.github.io/cufflinks/), for further analysis, including assembling transcripts and estimating their abundance[6]. Read counts were normalized to FPKM according to the gene length and total mapped reads. Differential gene expression was determined using the Single Cell Differential Expression (SCDE) toolset[7]. (Differential expression results in **Table S1** and SCDE coding in **Table S2**) Genes with a *P* value of less than 0.05 were considered "differentially expressed" and further analyzed for gene ontology (**Table S3**). Gene ontology and Kyoto Encyclopedia of Genes and Genomes (KEGG) pathway enrichment analyses were performed with DAVID informatics resources 6.7 of the National Institute of Allergy and Infectious Diseases (NIAID) and of the National Institutes of Health (NIH)(**Table S3**)[8,9]**.** RNA-seq data was analyzed with the assistance of Drs. Joshua Baller and John Garbe of the Minnesota Supercomputing Institute (MSI, University of Minnesota-Twin Cities).

Gene Cluster Analysis

Up-stream filtering of the data was done in the SingulaR package. A threshold of 1 FPKM was set as the limit of detection, which is the common threshold in the literature[10,11]. Outlier analysis was then performed in SingulaR with the identifyOutliers() command (See **Table S2**). No outliers were identified based on the gene expression. Over 12,000 genes with an FPKM >1 in at least one sample were used to obtain the HC analysis in **Fig. 2a, b**. Average linkage hierarchical clustering of gene expression intensity was performed using the Pearson distance to measure distance between gene and single cells. SingulaR (Fluidigm, San Francisco, CA) was used to compute and create the hierarchical clustering and principle component analysis plots.

Statistical Analysis

For comparison of DNA content and significant gene changes per chromosome a normal distribution was assumed and one-way analyses of variance and post-hoc test (Least Significant Difference, LSD) were used. To determine association between day of co-culture and phenotypic tendency to cardiomyocyte or mMSC (two by two table) the Yates corrected Chi-squared test was used. Data were analyzed with Microsoft Excel (Microsoft, Redmond, WA, USA). RNA-seq data was analyzed with the Cuffdiff or SingulaR programs.

Quantitative Real-Time Polymerase Chain Reactions (qRT-PCR)
Complementary DNA (cDNA) was synthesized following the instructions from the Maxima First Strand cDNA Synthesis Kit (cat# K1642, Thermo Scientific). The cDNA was amplified from the cDNA from the single-cell reaction performed in the Illumina chip. Primers of qRT-PCR were purchased from Biorad (Hercules, CA) and the sequence of *Gapdh* primers (FOR: CATGGCCTTCCGTGTTCCTA, REV: CCTGCTTCACCACCTTCTTGAT) was obtained from a previous report[12]. Primer efficiencies were extracted from RealPlex^2 software and verified with melting curves. The comparative C_t method[13] was employed to determine the relative changes in gene expression. Primer efficiencies were extracted from RealPlex^2 software and verified with melting curves. The comparative C_t method[13] was employed to determine the relative changes in gene expression. For comparison to the single-cell RNA-seq results, each gene's FPKM values were normalized to each cell's *Gapdh* FPKM value, which was then normalized to that of the control cells. *Nkx2-5* and *Nanog* were also probed for expression via qPCR analysis but were not detected in any cell probed. This may reflect the fact that PCR was conducted on residual cDNA from the RNA-seq procedure. In cases where qRT-PCR outcompetes RNA-seq in sensitivity, sequencing reads are limited and PCR amplification is conducted on an alternate cell of a given population.

Supplementary Figures

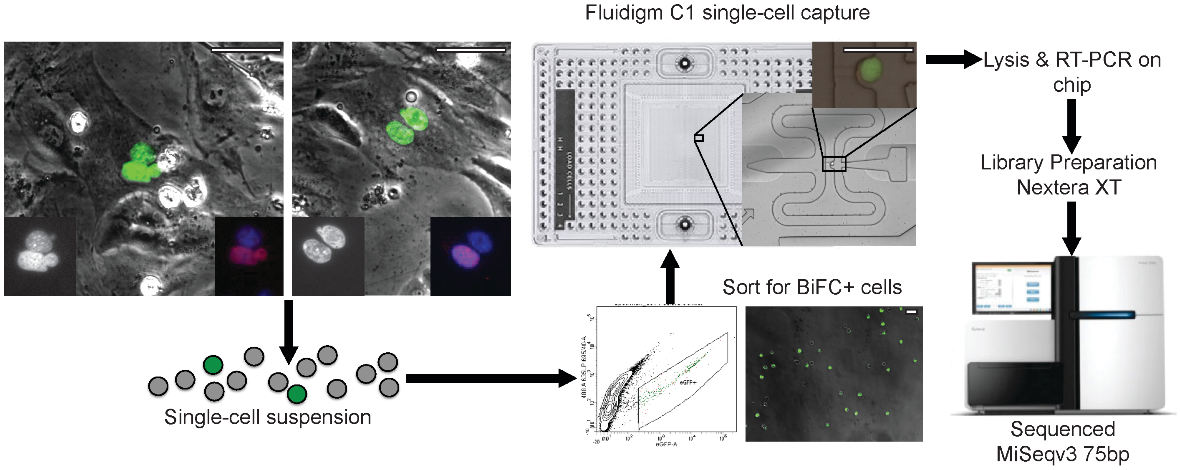

Figure S1. Workflow for identification, isolation and RNA-seq of mMSC-cardiomyocyte fusion products and associated controls, Related to Experimental Procedures. Fusion products were identified via detection of green fluorescence associated with the intact BiFC construct or dual color expression of both GFP and mCherry (and therefore cell fusion) via fluorescence microscopy, upper left panel. Merged image of bright field (to discern cell membranes) and BiFC (green). Scale bar = 50 µm. After identification, cells were removed from culture dishes and sorted for GFP^+ cells using FACS. Sorted cells were injected into the Fluidigm C1 chip and again visualized via fluorescence microscopy to ensure successful capture and to confirm cell viability (cells were also stained with DEAD cell viability assay). Cells of interest were lysed and RNA extraction and cDNA synthesis were performed on the chip. Library preparation followed using Illumina Nextera XT and then sequencing using MiSeqv3.

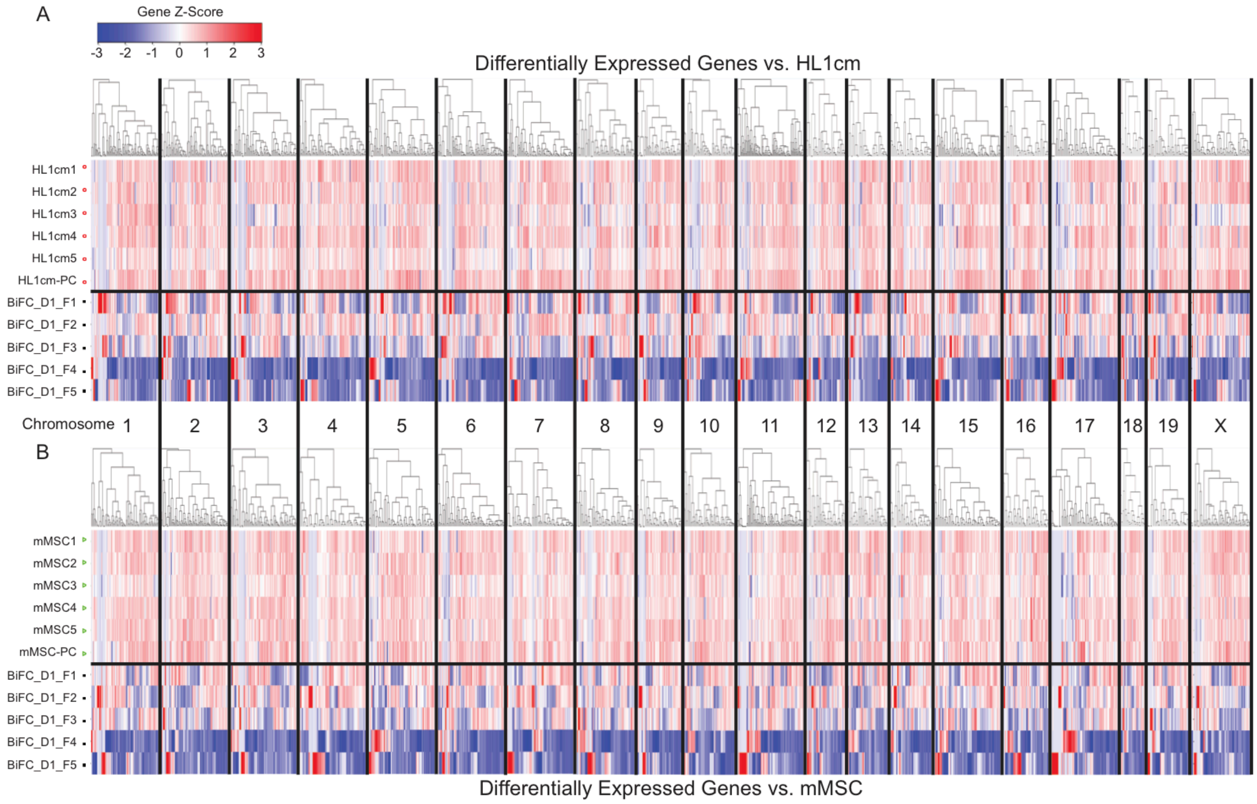

Figure S2. Hierarchical clustering of differentially expressed genes from cufflinks analysis of individual hybrids organized according to chromosome, Related to Figure 2. Differentially expressed genes of fusion products versus HL1cms (A) or mMSCs (B) were organized into chromosomal groups and hierarchical clustering was performed.

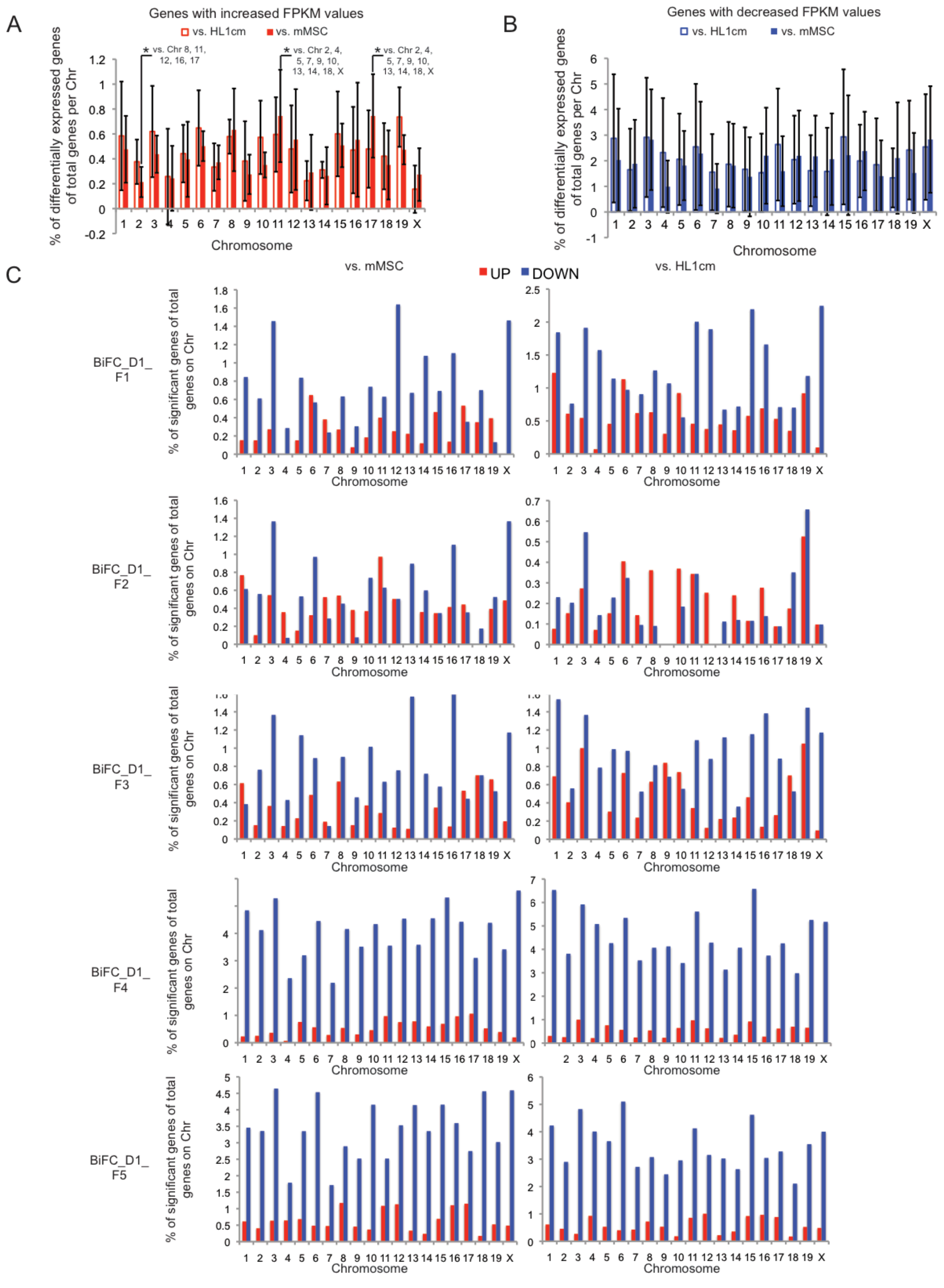

Figure S3. Differentially expressed genes of individual hybrids organized according to chromosome, Related to Figure 2. Total number of increased FPKM values (red) or decreased FPKM values (blue) from cufflinks analysis were normalized to the total number of genes on each chromosome for each fusion product (vs. HL1cm: open bars, vs. mMSC: closed bars). Increases FPKM values varied from chromosome to chromosome and between hybrids and, as shown in **Fig. S2**, occurred most often of chromosomes 2, 11 and 17. Decrease in FPKM values was universal and not chromosome specific. (**P* < 0.05 via LSD *post-hoc analysis*, mean $\pm$ SD)

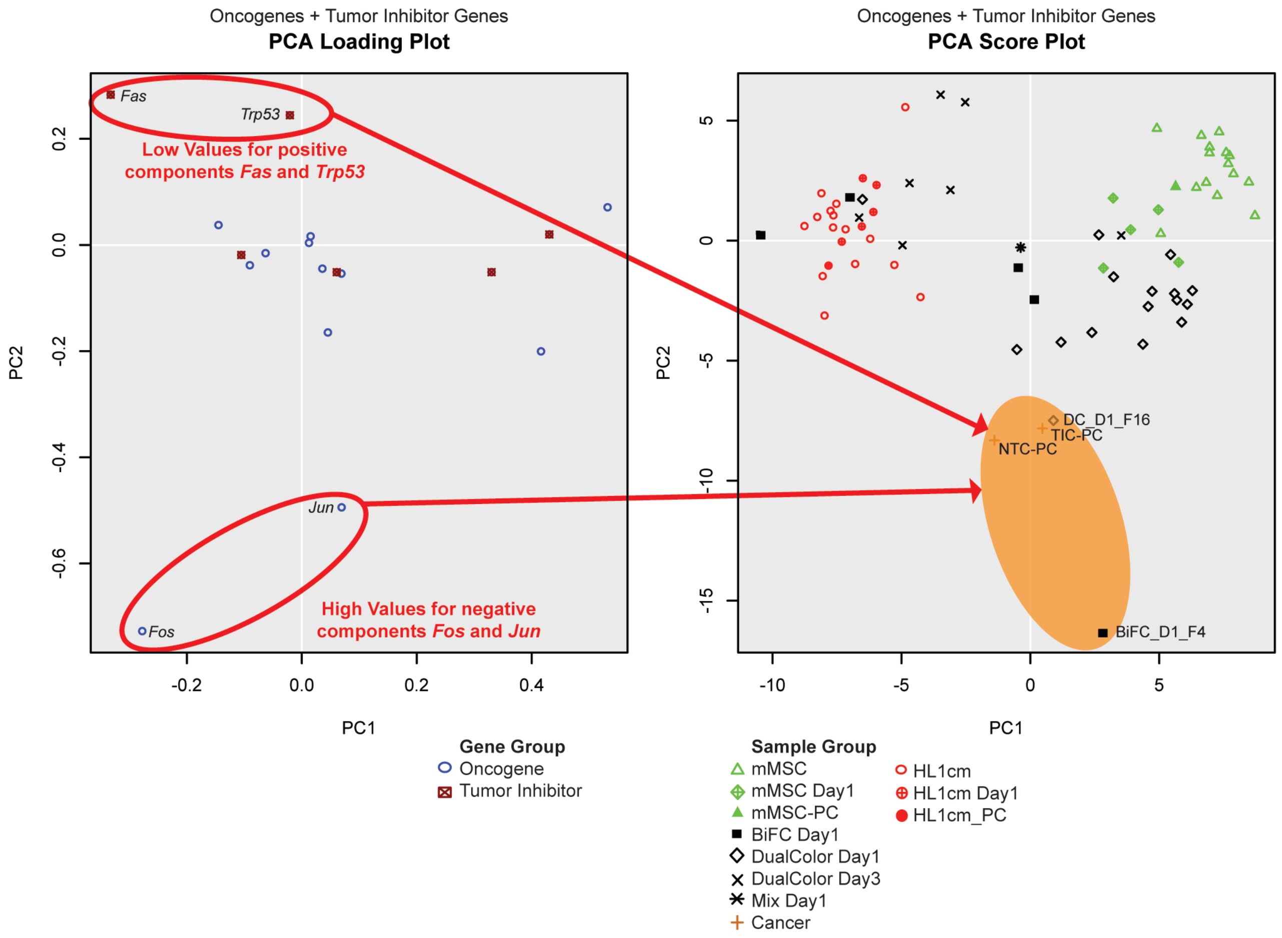

Figure S4. PCA loading plot and PCA Score plot for the cancer gene set, Related to Figure 4. The score plot is a summary of the relationship between single-cell samples (or population controls, PC). The loading plot is a summary of the genes and provides a means to interpret patterns seen in the score plot. Genes in the loading plot that fall far from zero on a PC axis are those that most significantly impact on the PC score of individual samples. In this case, *Fos* and *Jun* gene expression have a high negative contribution to the PC2 score, whereas *Fas* and *Trp53* expression have a high positive contribution to PC2. Therefore, if a cell has high expression of *Fos* and *Jun*, but low expression of *Fas* and *Trp53* it will have a negative PC2 value, as seen with BiFC_D1_F4 and DC_D1_F16 and the cancer populations. Interestingly, if BiFC_D1_F4 had lower expression of oncogenes *Fos* and *Jun* and increased expression of tumor suppressor genes *Trp53* and *Fas*, it would cluster more closely with the breast cancer cells on the PCA score plot.

Supplementary Tables

Table S1: Alignment statistics for single-cell with sequenced transcriptome, FPKM values and differentially expression genes of fusion products via SCDE, Related to Figure 2 and Figure 3

This first tab contains all of the alignment statistics for the captured fusion products and controls relative to the mouse genome. The number of aligned read pairs and the percent of concordant alignment (or reads with proper alignment) are listed for each single cell. The alignment data were generated from Tophat alignment results. The second tab is the single-cell RNA-seq expression data (FPKM values) for the fusion products (28 cells), HL1cm controls (20 cells and population control), mMSC controls (20 cells and population control) and the mixture of parental cells and fusion products (Mix_D1) in an excel file as discussed in Figures 2, 3, and 4. Also included is the FPKM values for breast cancer populations found in the literature and utilized for comparison in Figure 4E, F (GSE41286)[14]. The remainder of the file contains all of the differentially expressed genes detected with the SCDE toolset between the fusion products with unique transcriptomes and the parental controls.

Table S2: SingulaR and SCDE scripts used to analyze single-cell RNA-seq data, Related to Experimental Procedures

This file contains the SingulaR and SCDE script used with the single-cell RNA-seq data to create the hierarchical clustering, principal component analysis and gene differential.

Table S3: Gene ontology and KEGG pathway enrichment analysis, Related to Figure 3.

This file contains all of the differentially expressed genes of the combined fusion products with unique transcriptomes compared to either parental cell, but organized into lists used for gene ontology on the first tab. The file is organized into differentially expressed genes with increased FPKM values versus mMSCs, genes with decreased FPKM values versus mMSCs, genes with increased FPKM values versus HL1cm and genes with decreased FPKM values versus HL1cm. The remaining tabs contain the gene ontology and KEGG pathway enrichment analysis that was performed using DAVID informatics Resources 6.7 for increase or decrease in FPKM values of the differentially expressed gene lists for fusion products with unique transcriptomes versus both parental cell types as identified in the SCDE analysis.

Table S4: Genes lists, Related to Figure 4.

This file contains the genes associated with each gene list (cardiac[15-18], MSC[19-26], and cancer[27] from gene ontology). Included in this file are the gene symbol, full gene name, functional group and source for inclusion. The second tab contains expanded gene lists (Differentiation, Stemness, and Cardiac) compiled during analysis of the samples.

References

1. Trivedi, P. & Hematti, P. Simultaneous generation of CD34+ primitive hematopoietic cells and CD73+ mesenchymal stem cells from human embryonic stem cells cocultured with murine OP9 stromal cells. *Exp Hematol* **35**, 146-154 (2007).
2. Steinhelper, M. E. *et al.* Proliferation in vivo and in culture of differentiated adult atrial cardiomyocytes from transgenic mice. *Am J Physiol* **259**, H1826-1834 (1990).
3. Delcarpio, J. B., Lanson, N. A., Jr., Field, L. J. & Claycomb, W. C. Morphological characterization of cardiomyocytes isolated from a transplantable cardiac tumor derived from transgenic mouse atria (AT-1 cells). *Circ Res* **69**, 1591-1600 (1991).
4. Zhang, P. *et al.* Interactions among measles virus hemagglutinin, fusion protein and cell receptor signaling lymphocyte activation molecule (SLAM) indicating a new fusion-trimer model. *J Biochem Mol Biol* **38**, 373-380 (2005).
5. Lin, H. P., Vincenz, C., Eliceiri, K. W., Kerppola, T. K. & Ogle, B. M. Bimolecular fluorescence complementation analysis of eukaryotic fusion products. *Biol Cell* **102**, 525-537 (2010).
6. Trapnell, C. *et al.* Differential gene and transcript expression analysis of RNA-seq experiments with TopHat and Cufflinks. *Nat Protoc* **7**, 562-578 (2012).
7. Kharchenko, P. V., Silberstein, L. & Scadden, D. T. Bayesian approach to single-cell differential expression analysis. *Nat Methods* **11**, 740-742 (2014).
8. Huang da, W., Sherman, B. T. & Lempicki, R. A. Bioinformatics enrichment tools: paths toward the comprehensive functional analysis of large gene lists. *Nucleic Acids Res* **37**, 1-13 (2009).
9. Huang da, W., Sherman, B. T. & Lempicki, R. A. Systematic and integrative analysis of large gene lists using DAVID bioinformatics resources. *Nat Protoc* **4**, 44-57 (2009).
10. Ramskold, D., Wang, E. T., Burge, C. B. & Sandberg, R. An abundance of ubiquitously expressed genes revealed by tissue transcriptome sequence data. *PLoS Comp Biol* **5**, e1000598 (2009).
11. Henley, B. M. *et al.* Transcriptional regulation by nicotine in dopaminergic neurons. *Biochem Pharmacol* **86**, 1074-1083 (2013).
12. Tsujita, Y. *et al.* Nuclear targeting of Akt antagonizes aspects of cardiomyocyte hypertrophy. *Proc Natl Acad Sci U S A* **103**, 11946-11951 (2006).
13. Livak, K. J. & Schmittgen, T. D. Analysis of relative gene expression data using real-time quantitative PCR and the 2(-Delta Delta C(T)) Method. *Methods* **25**, 402-408 (2001).
14. Feng, W. *et al.* Targeting unique metabolic properties of breast tumor initiating cells. *Stem Cells* **32**, 1734-1745 (2014).
15. Olson, E. N. Gene regulatory networks in the evolution and development of the heart. *Science* **313**, 1922-1927 (2006).
16. Olson, E. N. & Schneider, M. D. Sizing up the heart: development redux in disease. *Genes Dev* **17**, 1937-1956 (2003).
17. Abu-Issa, R. & Kirby, M. L. Heart field: from mesoderm to heart tube. *Annu Review Cell Dev Biol* **23**, 45-68 (2007).
18. Mummery, C. L. *et al.* Differentiation of human embryonic stem cells and induced pluripotent stem cells to cardiomyocytes: a methods overview. *Circ Res* **111**, 344-358 (2012).
19. Tanaka, T. S. *et al.* Gene expression profiling of embryo-derived stem cells reveals candidate genes associated with pluripotency and lineage specificity. *Genome Res* **12**, 1921-1928 (2002).
20. Funari, V. A. *et al.* Cartilage-selective genes identified in genome-scale analysis of non-cartilage and cartilage gene expression. *BMC Genomics* **8**, 165 (2007).
21. Kolf, C. M., Cho, E. & Tuan, R. S. Mesenchymal stromal cells. Biology of adult mesenchymal stem cells: regulation of niche, self-renewal and differentiation. *Arthritis Res Ther* **9**, 204 (2007).

22. Zaidi, M. Skeletal remodeling in health and disease. *Nat Med* **13**, 791-801 (2007).
23. Ng, K. K., Thatte, H. S. & Spector, M. Chondrogenic differentiation of adult mesenchymal stem cells and embryonic cells in collagen scaffolds. *J Biomed Mater Res A* **99**, 275-82 (2011).
24. Park, J. S. *et al.* Mechanobiology of mesenchymal stem cells and their use in cardiovascular repair. *Front Biosci* **12**, 5098-5116 (2007).
25. James, C. G. *et al.* Genome-wide analyses of gene expression during mouse endochondral ossification. *PLoS One* **5**, e8693 (2010).
26. Brambrink, T. *et al.* Sequential expression of pluripotency markers during direct reprogramming of mouse somatic cells. *Cell Stem Cell* **2**, 151-159 (2008).
27. Johansson, F. K. *et al.* Identification of candidate cancer-causing genes in mouse brain tumors by retroviral tagging. *Proc Natl Acad Sci U S A* **101**, 11334-11337 (2004).
